# Six-Month Progression of Massive Left Ventricular Pseudoaneurysm

**DOI:** 10.1016/j.cjco.2024.01.007

**Published:** 2024-02-01

**Authors:** Nick S.R. Lan, Eric Slimani, Aife O’Brien, Lawrence Dembo, Kalilur Anvardeen, Kaitlyn Lam

**Affiliations:** a Medical School, The University of Western Australia, Crawley, Perth, Western Australia, Australia; b Advanced Heart Failure and Cardiac Transplant Service, Fiona Stanley Hospital, Murdoch, Perth, Western Australia, Australia; c Department of Cardiology, Fiona Stanley Hospital, Murdoch, Perth, Western Australia, Australia; d Department of Cardiothoracic Surgery and Transplantation, Fiona Stanley Hospital, Murdoch, Perth, Western Australia, Australia; e Medical School, Curtin University, Bentley, Perth, Western Australia, Australia

A 59-year-old male patient was referred to our centre after a scheduled outpatient echocardiogram demonstrated that he had a left ventricular (LV) pseudoaneurysm. The patient reported chest pain and dyspnea on exertion. He had a history of delayed presentation myocardial infarction (MI) 6 months prior, with negative cardiac troponin at presentation. At the time of MI, he was a smoker but had no other past medical history of note. Percutaneous intervention, with placement of a drug-eluting stent to a severe mid left anterior descending artery lesion was performed, and thrombotic occlusion of the principal diagonal artery was medically managed. Echocardiography at the time of MI presentation was diagnosed as an LV aneurysm (Fig. 1A, **green arrow**), and prophylactic warfarin was initiated in conjunction with dual-antiplatelet therapy for 1 month, followed by warfarin and single-antiplatelet therapy thereafter.

**Figure 1:**
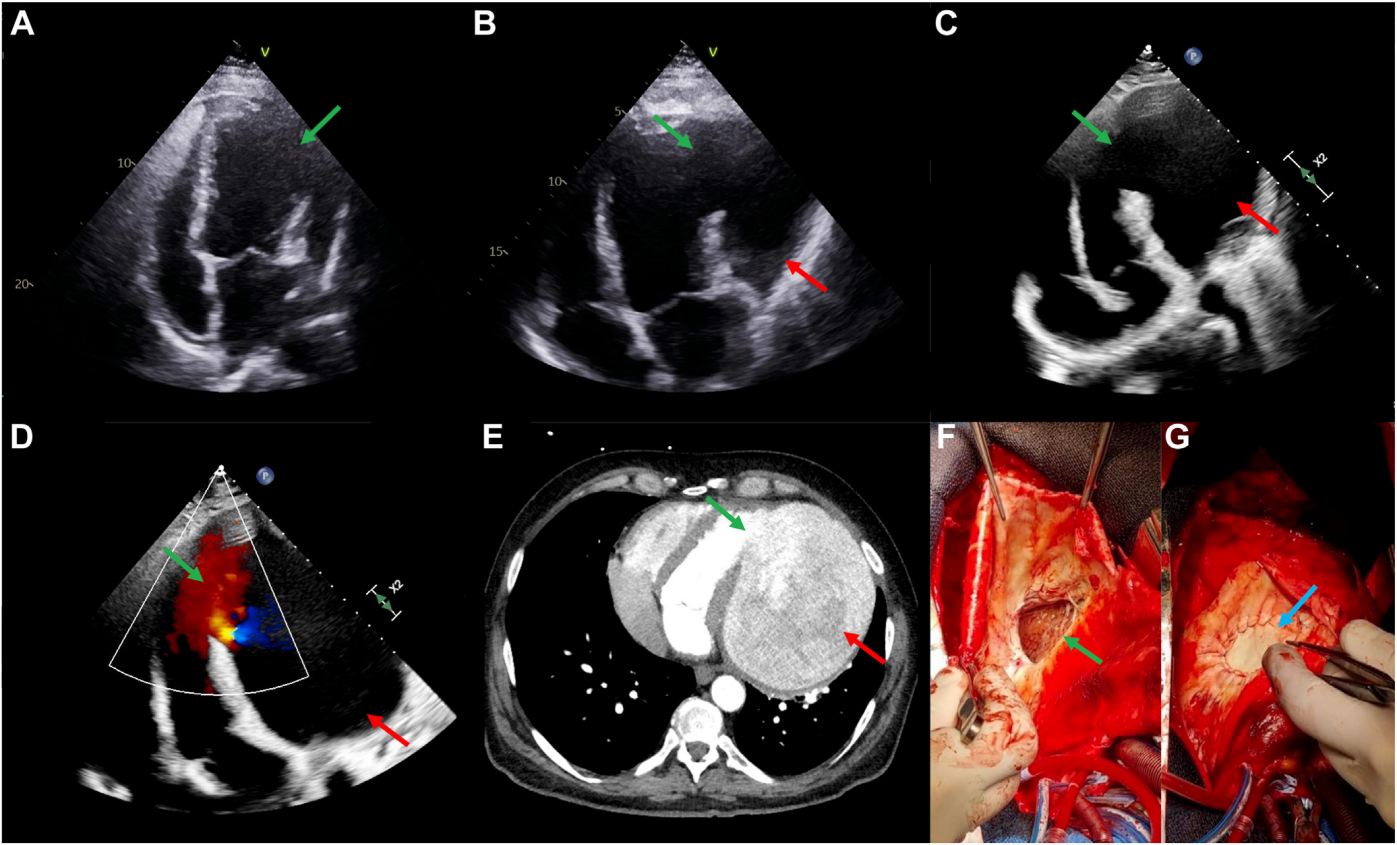
Left ventricular wall rupture (**green arrows**) and a pseudoaneurysm (**red arrows**) are shown on a transthoracic apical 4-chamber view (**A**) at the time of myocardial infarction, (**B**) 4 months later, and (**C**) 6 months later. (**D**) Colour flow Doppler echocardiography shows flow between the left ventricle and the pseudoaneurysm. (**E**) Cardiac computed tomography at 6 months shows the left ventricular wall rupture with a massive pseudoaneurysm. Intraoperative photography shows (**F**) the defect and (**G**) patch repair (**blue arrow**).

Echocardiography 4 months post-MI showed an LV pseudoaneurysm (Fig. 1B, **red arrow**) from anterior and anterolateral wall rupture (Fig. 1B, **green arrow**) measuring 6 × 11 cm. This was not intervened on at the time, as it was initially thought to be a worsening LV aneurysm. At 6 months post-MI, echocardiography was performed, and the patient was urgently referred to our centre. Echocardiography 6 months post-MI (Video 1 , view video online) showed continued enlargement of the pseudoaneurysm (Fig. 1C, **red arrow**) from wall rupture (Fig. 1C**, green arrow**), measuring 15 × 11 cm, with a pseudoaneurysm neck measuring 5.5 cm. Colour flow Doppler (Fig. 1D, **green arrow;** Video 2 , view video online) and Definity contrast (Video 3 , view video online) demonstrated flow from the left ventricle into the pseudoaneurysm (Fig. 1D, **red arrow**).

Urgent cardiac computed tomography (Video 4 , view video online) 6 months post-MI demonstrated a 15 x 11 x 16 cm contrast-filled outpouching (Fig. 1E**, red arrow**) arising from the mid-to-distal LV anterolateral wall (Fig. 1E, **green arrow**), consistent with contained rupture following left anterior descending territory MI. Surgical repair of the defect (Fig. 1F**, green arrow**) was urgently performed, with a 6 × 8 cm bovine pericardial patch (Fig. 1G, **blue arrow**) sutured in position with double-layer continuous prolene. The remnants of the pseudoaneurysm sac were closed over the patch with double-layer continuous prolene. Histopathology showed fibrous connective tissue with adherent clot, in keeping with LV pseudoaneurysm. The postoperative course was uncomplicated, with an intensive care unit length of stay of 4 days. Prior to hospital discharge, medical therapy for heart failure was recommenced, and an implantable cardioverter defibrillator was inserted for primary prevention. The patient was discharged on day 20 post-repair.

LV pseudoaneurysm post-MI occurs when cardiac rupture is contained by pericardial adhesions, and it is an uncommon complication, reported in < 0.1% of patients.^1^^,^^2^ Differentiating LV pseudoaneurysms from true aneurysms can be challenging, but it is important, as urgent surgery is the treatment of choice for pseudoaneurysms, owing to the high risk of rupture (30%-45%) and mortality.^1^^,^^2^ LV pseudoaneurysms have a narrow neck and are often located in the inferior or lateral wall.^1^ Using Doppler echocardiography, turbulent flow may be detected at the neck of the pseudoaneurysm or within the pseudoaneurysm itself.^3^ On the other hand, true LV aneurysms have a wide neck, are made of thin, scarred, and nonfunctional myocardium, and often involve the anterior or apical walls.^2^ Few reports have been made on the natural history of conservatively managed disease.^2^ In this unique case, the patient survived 6 months with an enlarging, massive pseudoaneurysm.

**Novel Teaching Points**

• Differentiating true LV aneurysms from pseudoaneurysms is important; the latter typically have a narrow neck. A high index of suspicion is required, and multi-modality imaging can help make the diagnosis.
• Few reports have been made of conservatively managed LV pseudoaneurysm, as the treatment of choice is urgent surgical intervention, owing to the risk of disease progression and rupture with conservative management.

## Supplementary Material

Video 1 Transthoracic apical four chamber view demonstrating the left ventricular pseudoaneurysm from anterior/anterolateral wall rupture.

Video 2 Transthoracic apical four two view with colour flow Doppler demonstrating flow between the left ventricle and the pseudoaneurysm.

Video 3 Transthoracic apical four chamber view demonstrating Definity contrast entering the pseudoaneurysm from the left ventricle.

Video 4 Cardiac computed tomography transverse view demonstrating the contrast-filled pseudoaneurysm arising from the mid-to-distal left ventricular anterolateral wall.
